# Wedge resection combined with 3D-printed polycaprolactone mesh for caudal septal deviation

**DOI:** 10.1186/s40463-023-00677-y

**Published:** 2023-10-24

**Authors:** Jee Won Moon, Seok-Youl Choi, Su-Jong Kim, Jae-Min Shin, Il-Ho Park

**Affiliations:** 1 Department of Otorhinolaryngology-Head and Neck Surgery, Guro Hospital, Korea University College of Medicine, 08308 Seoul, South Korea; 2 Upper Airway Chronic Inflammatory Diseases Laboratory, Korea University, College of Medicine, Seoul, South Korea; 3 Medical Device Usability Test Center, Korea University, College of Medicine, Seoul, South Korea

**Keywords:** Wedge resection, Polycaprolactone, Caudal septal deviation, Septoplasty, 3-D printing, Nasal mesh

## Abstract

**Background:**

Biocompatibility and stability of three-dimensional printed polycaprolactone mesh grafts for nasal surgery are proven in both animal and human models. However, their safety and durability as batten grafts for caudal septal deviation has not been documented. This study was designed to investigate the efficacy and safety of three-dimensional printed polycaprolactone mesh batten graft in septoplasty using the wedge resection technique for the correction of caudal septal deviation.

**Methods:**

This retrospective study reviewed the medical records of 20 patients aged ≥ 18 years with caudal septal deviation who underwent septoplasty with wedge resection and three-dimensional printed polycaprolactone mesh graft from a tertiary medical center in South Korea, between December 1, 2019 and May 31, 2021. Those without nasal obstruction before surgery or with a short follow-up period (< 28 days) were excluded from the survey analysis.

**Results:**

Of the 20 patients (mean age, 48.0 [range, 19–65] years), 17 (85.0%) were male, and three (15.0%) were female. A significant change was noted in the mean nasal obstruction symptom evaluation score (68.2 vs. 15.0, *P* < .001) in the 17 patients included in the analysis. Postoperative endoscopic evaluation revealed a straight septum in 19/20 (95.0%) patients, and no complications were noted in the postoperative follow-up period of up to 364 days.

**Conclusions:**

The three-dimensional printed polycaprolactone nasal mesh is safe and provides adequate support to resist the intrinsic memory of the cartilage of the caudal septum. In addition to nasal surgeries, it has great potential as a graft in other reconstructive surgeries.

*Trial registration* Retrospectively registered.

## Background

Nasal septal deviation, a condition in which the nasal septum is displaced to one side, can occur due to various causes, and affected patients mainly complain of nasal obstruction. In otorhinolaryngology, septoplasty is frequently performed to correct septal deviation without external deformities. However, management of caudal septal deviation, which causes narrowing of the external valve area with involvement of the L-strut, is challenging because the intrinsic memory of the cartilage is difficult to overcome and external nose deformities are more likely to occur after surgery [1–3].

In the past, several techniques have been developed for correction of caudal septal deviation without dislocation or subluxation of the anterior nasal spine; however, multiple combinations of methods are often needed to successfully correct the deviation. The surgical outcomes of incisional or excisional techniques are dependent on the secondary healing process, due to which the outcomes are inconsistent [3–7]. Meanwhile, the cut-and-suture technique (C&S technique) permits intraoperative verification of the straightened septum and is also effective in reducing cartilage length and providing support to the L-strut through the overlapping cartilage [3]. However, as the nasal septal cartilage in Asians is very thin compared to that in other races, batten grafts are frequently used for extra support [8].

Batten grafts are usually harvested from the septal cartilage, bone, or ethmoid plate during the procedure, but autografts are often limited in size or strength and sometimes even unavailable in revision surgeries [1, 2, 9]. Consequently, various allografts have been studied as substitutes for autografts, but increased risks of infection, graft displacement or functional failure exist [10, 11]. Recently, 3-dimensional (3-D) printing is emerging as a prospective technology in the field of reconstructive or facial surgeries, and it has been proven that a 3-D printed mesh can provide enough support for the nasal septum postoperatively [12]. Through 3-D printing, we can produce individualized or standardized grafts that suit patients’ needs, with regard to size, width, and structure. Polycaprolactone (PCL) is a bioabsorbable polyester that is degraded by hydrolysis in physiologic conditions, and can be used as a raw material for 3-D printing. Moreover, the biocompatibility and stability of a 3-D printed PCL mesh in nasal surgeries has been proven in previous studies [10, 13–15]. Through adopting a reliable and sturdy PCL graft for the septum, we could minimize the overlapping of the caudal cartilage, enabling end-to-end anastomosis by resecting a wedge-shaped segment of the most-bent portion of the caudal cartilage (Wedge resection technique). Therefore, in this study, we investigated the surgical outcomes of septoplasty using the wedge resection technique along with PCL mesh implantation for the correction of caudal septal deviation.

## Methods

### Patient selection

We retrospectively reviewed the medical records of patients who underwent septoplasty with wedge resection and PCL mesh graft for caudal septal deviation at the Korea University Guro Hospital between December 1, 2019 and May 31, 2021. All patients were at least 18 years old at the time of surgery, and those with allergic rhinitis (AR) underwent surgery only when nasal obstruction persisted despite medical treatment. All surgeries were performed by a single surgeon (IH Park), and patients without preoperative complaints associated with nasal obstruction were excluded from the analysis. The follow-up period was defined as the number of days from the date of surgery to the date of the last outpatient clinic visit. This study was approved by the Korea University Medical Center Institutional Review Board (2021GR0256).

### Surgical procedure

**Fig. 1:**
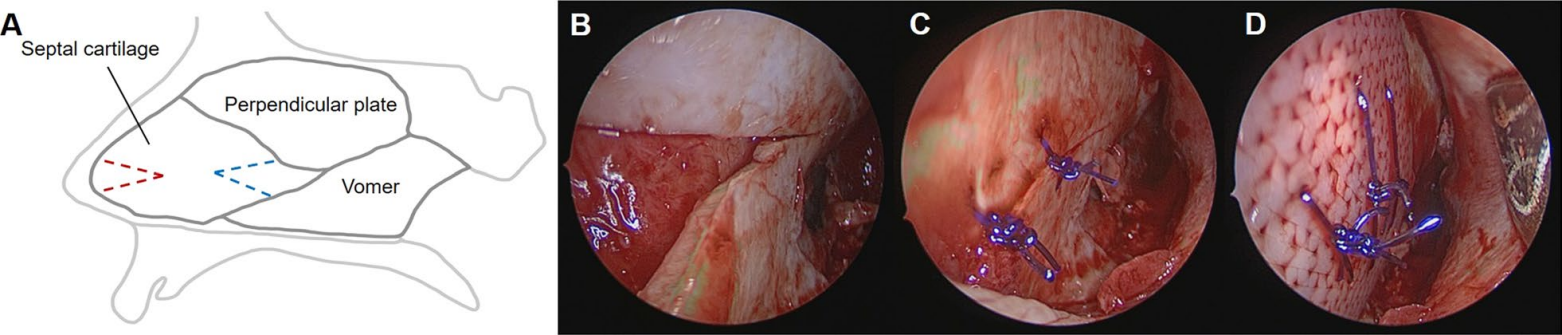
Wedge resection with application of PCL mesh. **A** Schematic drawing of wedge resection (red or blue dotted line). **B** Wedge resection of the most-bent portion of the L-strut. **C** End-to-end anastomosis of the remaining septal cartilage. **D** Trimming, positioning, and suturing of the PCL mesh. PCL, polycaprolactone

Under general anesthesia, local anesthetic infiltration was performed using 1% lidocaine with 1:200,000 epinephrine along the septal wall. A hemitransfixion incision was made at the left caudal end of the septal cartilage, followed by bilateral mucosal flap elevation. The junctions between the cartilage and the perpendicular plate and maxillary crest were separated, and the mucoperiosteum on the opposite side was elevated. The deviated bony and cartilaginous portions were removed with preservation of the L-strut. A wedge-shaped segment of the most-bent caudal portion of the L-strut was resected (wedge resection technique), and the upper and lower parts of the remaining septal cartilage were sutured with 4–0 PDS. Then, a 0.5-mm-thick PCL mesh was trimmed, positioned, and sutured with 4–0 PDS to provide additional support to the L-strut (Fig. 1). The incision site was sutured with 3–0 Vicryl, and inferior turbinoplasty was performed when needed. After bilateral silastic sheet insertion, nasal packing was performed using a rapid rhino.

### Polycaprolactone mesh

A 0.5-mm-thick polycaprolactone mesh, with a line width and the triangular pore size of 500 μm each, was irradiated with gamma rays (15 kGy) after fabrication through 3-D printing. The PCL mesh (T&R Biofab Co Ltd, Siheung, Korea) used in this study was approved for manufacturing and marketing by the Korean Ministry of Food and Drug Safety.

### Subjective evaluation

Pre- and postoperative nasal obstruction symptom evaluation (NOSE) surveys were conducted for all patients, and preoperative epistaxis or headache were scored (0–10) using a visual analog scale (VAS). Subjective improvement in nasal obstruction was surveyed using a 5-point Likert scale at 4–8 weeks postoperatively. Missing data were obtained through telephonic surveys.

### Objective evaluation

Pre- and postoperative endoscopic evaluations were performed by two experienced otolaryngologists (postoperative evaluation performed at 4–8 weeks postoperatively), and the change in the septum was evaluated as follows: straight, improved with residual deviation, or no change. During the follow-up period, the patients were inspected thoroughly to identify external nose deformities.

### Post-operative care

Bilateral nasal packing was removed on postoperative day 2, and silastic sheets were removed approximately 2 weeks after the surgery at the outpatient clinic visit. Medical treatment was continued in patients who tested positive for allergy (either in the skin prick test or ImmunoCAP; Thermo Fisher Diagnostics).

### Statistical analysis

A paired t-test was conducted to compare preoperative and postoperative NOSE and VAS scores, and the Wilcoxon signed rank test was conducted for sub-analysis based on the presence of AR. All statistical analyses were conducted using SPSS ver. 22.0, and *P* < 0.05 was considered statistically significant.

## Results

### Patient characteristics

A total of 20 patients (17 male and 3 female) underwent septoplasty with wedge resection and PCL mesh graft for caudal septal deviation without subluxation or dislocation of the anterior nasal spine. The mean age of the patients at the time of surgery was 48.0 years, and the mean follow-up period was 112.8 days (Table 1). Ten patients tested positive for allergy, three patients had a history of nasal bone fracture, and one patient underwent revision septoplasty. Concurrent procedures were as follows: four patients underwent endoscopic sinus surgery, 16 patients underwent inferior turbinate surgery, and dorsum and valve surgery were performed in 1 patient each (Table 2).

**Table 1:** Patient characteristics

Characteristics	n(%), N = 20
Age, mean (range), years	48.0 (19–65)
Sex	
Male	17 (85.0)
Female	3 (15.0)
Allergic rhinitis	10 (50.0)
Previous history	
Nasal bone fracture	3 (15.0)
Septoplasty	1 (5.0)
Mean follow-up period, mean (range), days	112.8 (27–349)

**Table 2:** Concurrent procedures and postoperative complications

	n (%), N = 20
Concurrent procedures	48.0 (19–65)
Endoscopic sinus surgery	4 (20.0)
Turbinate surgery	17 (85.0)
Dorsum surgery	3 (15.0)
Valve surgery	10 (50.0)
Post-op complication	0 (0.0)

### Comparison of the pre-operative and post-operative NOSE, and VAS scores

**Fig. 2:**
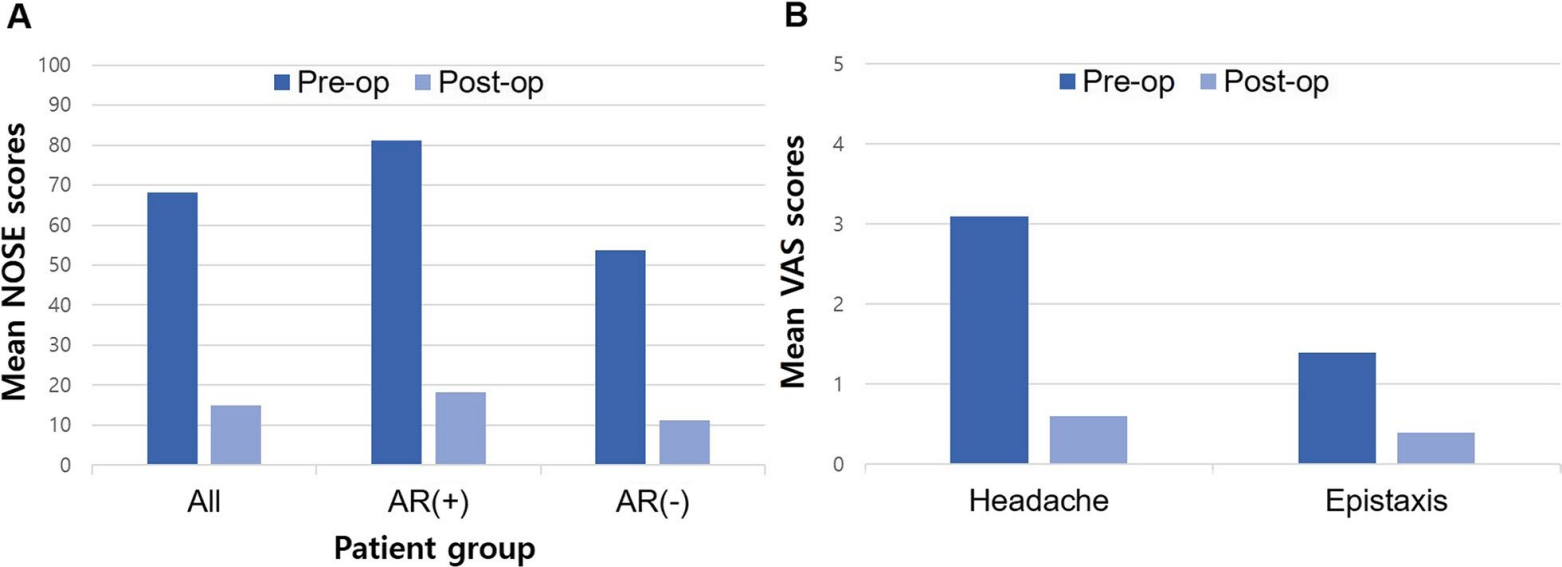
Pre/post operative scores. **A** Comparison of the pre/post operative NOSE scores. **B** Comparison of the pre/post operative VAS scores. NOSE, nasal obstruction symptom evaluation; VAS, visual analogue scale; AR(+), allergic rhinitis group; AR(–), non-allergic rhinitis group

Of the 20 patients, three were excluded from the final survey analysis due to the absence of nasal obstruction before surgery or a short follow-up period. The mean preoperative NOSE score for 17 patients was 68.2, which decreased to 15.0 postoperatively (*P* < 0.001). In the nine patients with AR, the mean NOSE score dropped from 81.1 to 18.3 (*P* < 0.001) and in the eight patients without AR, the mean NOSE score dropped from 53.8% to 11.3 (*P* = 0.001). The mean VAS score for headache and epistaxis dropped from 3.1 to 0.6 (*P* = 0.009) and 1.4 to 0.4 (*P* = 0.114), respectively (Fig. 2).

### Subjective change of nasal obstruction

**Fig. 3:**
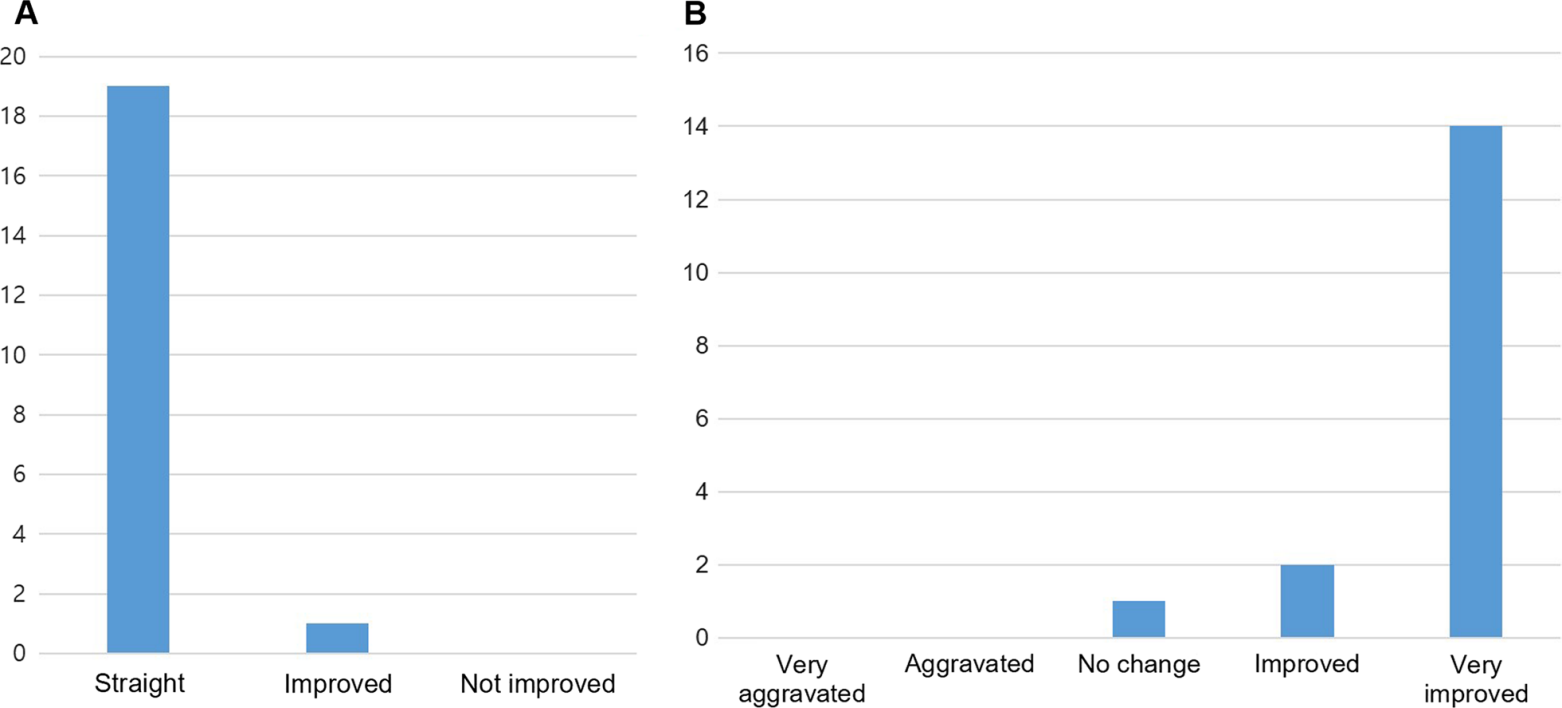
Postoperative evaluation. **A** Endoscopic evaluation of surgical outcomes. **B** Subjective change in nasal obstruction

Of the 17 patients, subjective changes in nasal obstruction after surgery based on a 5-point Likert scale were as follows: greatly improved, 14 patients; improved, 2 patients; and no change, 1 patient (Fig. 3-B).

### Postoperative endoscopic evaluation

**Fig. 4:**
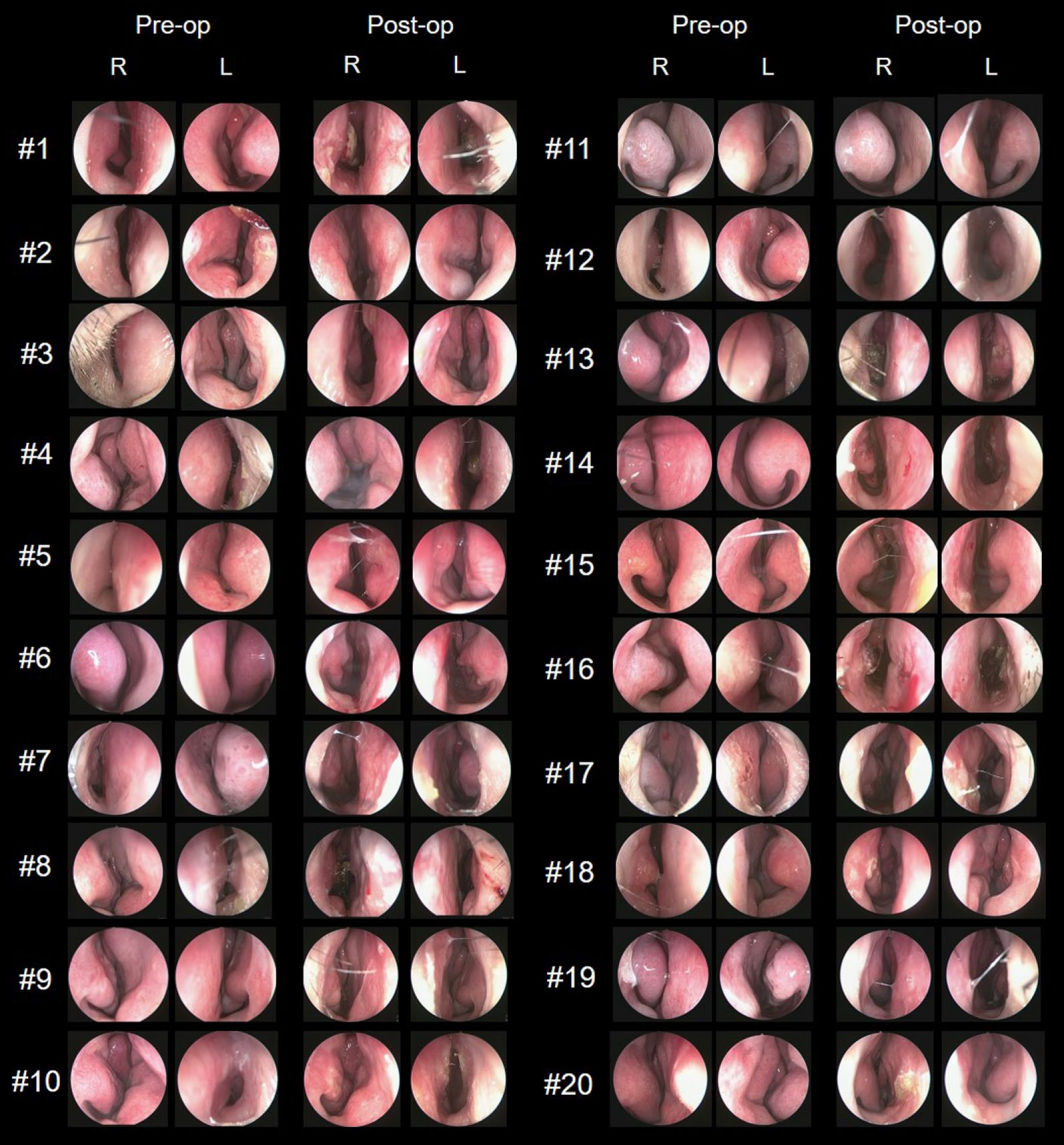
Pre-and postoperative nasal endoscopy images of patients

Postoperative endoscopic evaluation revealed a straight septum in 19 patients; 1 patient showed improvement, but with residual septal deviation (Figs. 3-A, 4).

### Postoperative complications

During the postoperative follow-up period of up to 349 days, no major complications such as septal hematoma, inflammation, or external nose deformity were noted. Complications related to the septal implant or suture, such as inflammation, signs of irritation on the septum, or implant extrusion, were not observed in any patient (Table 2, Fig. 4).

## Discussion

Severe caudal cartilage deviation frequently involves the L-strut, and symptom improvement in patients with caudal septal deviation is not likely to be achieved without direct modification of the L-strut. However, most surgeons inevitably adopt defensive measures to avoid postoperative cosmetic issues. Surgical techniques such as the suture technique, tongue-in-groove, and swinging door were invented to correct caudal septal deviation, but these techniques cannot effectively manage the excess cartilage length, which is the main problem in anteroposterior caudal septal deviation [16].

The C&S technique, which effectively reduces excess cartilage length without affecting the original tip height, was introduced by Jang et al. in 2009 [3]. However, the superposition of the cartilage on the remaining natural curvature in the C&S technique may reduce the cross-sectional area of the nasal cavity in patients with severe caudal septal deviation. To minimize the effect of the overlapping cartilage, complete removal of the deviated portion and end-to-end anastomosis are required. Hosokawa et al. introduced modified technique that connects the cartilage bank to the anterior nasal spine to avoid adding batten grafts, but the risk of pollybeak deformity persists if the cartilage is cut excessively [17]. Jeon et al. devised a triangular excision and submucosal rejoining technique using the natural fracture line formed on the quadrangular cartilage, but it is of limited use when the fracture line does not involve the most-bent portion of the caudal septum [18]. The wedge resection technique used in this study is not only useful for reducing the cartilage length, but also effective in eliminating septal curvature and internal cartilage memory by removing most of the deviated portion of the cartilage. The upper and lower margins of the remaining cartilage are sutured together to reconstruct the attachment, and the increased concern for a weakened L-strut can be alleviated by adopting batten grafts, such as the bioabsorbable mesh used in this study.

Cartilaginous or bony autografts are often used as batten grafts to reinforce the modified septal cartilage. However, autografts large and strong enough to support the deviated septum cannot always be obtained from the patient. Moreover, harvesting autografts can cause loss of tip support [13]. Cartilaginous grafts are more likely to cause saddle nose or external deformity due to their inability to bear nasal weight, and bony grafts need drilling to be sutured on the residual cartilage, which is a bothersome procedure for surgeons [2, 5]. Thus, allografts that can be surgically manipulated and have sufficient strength to withstand nasal weight are needed to replace autografts.

In 2016, Kang et al. implanted a PDS-like bioabsorbable plate as a batten graft for caudal septal deviation, and no major complications were reported in the enrolled patients [1]. However, in our experience, the PDS-like plate is not strong enough to support the modified septum. Other alloplastic implants have been used for the reconstruction of the septum, but most of the materials have major drawbacks in terms of ease of surgical manipulation [10].

PCL has been approved by the U.S. Food and Drug Administration for use in the human body, specifically as a drug delivery device, suture, or adhesion barrier. In a previous animal study, a 3-D printed PCL mesh showed efficacy in maintaining tip refinement without material-related complications, and its micropores allowed for fibroblastic growth and fibrovascular permeation from the surrounding host tissue [13]. The 3-D printed PCL mesh is more affordable and allows better surgical manipulation than other biodegradable plates. In addition, its porous structure contributes to the reconstruction of the damaged septum by allowing migration of the nearby normal tissue, which helps enhance the stability of the modified septum. The compressive stiffness of PCL implants is similar to that of cartilage, which means that the mechanical properties of the PCL mesh correspond with those of the cartilage [19, 20]. Additionally, the 0.5-mm-thick mesh used in this study did not reduce the cross-sectional area of the nasal cavity, preventing possible secondary nasal obstruction caused by the batten graft. To our knowledge, there is no marketed PCL mesh for reconstructive surgery or graft in USA or Europe so far. However, the research is ongoing for its use on stress urine incontinence or pelvic prolapse with satisfying results, so we expect the PCL mesh soon to be available worldwide [21, 22].

A previous study evaluating the mechanical properties of PCL mesh also reported surgical outcomes in caudal septal deviation [10]. However, the techniques applied to the caudal septum before implanting the mesh were heterogeneous, and the overall severity of deviation before surgery could not be assessed. Most of the patients enrolled in the present study had severe caudal septal deviation almost reaching the lateral nasal wall (Fig. 4), and caudal deviations were mostly modified using the wedge resection technique (Table 2). Moreover, significant symptomatic improvements were noted, and postoperative endoscopy confirmed a straight septum in most of the patients without any complications (Figs. 2, 3, 4). None of the patients presented with re-deviation or external deformity of the nose during the follow-up period of up to 1 year after the surgery, which demonstrates the long-term stability of the PCL mesh. In addition, no inflammatory signs or foreign body reactions to the PCL mesh, which is known to be degraded in the body within 2–3 years after implantation, were noted during the observation period, proving its biocompatibility in the human body. Meanwhile, patient no. 6 complained of persistent nasal obstruction after surgery, even though postoperative endoscopy confirmed a straight septum and no visible anatomic cause for obstruction could be identified.

## Limitations

This study has some limitations. First, it lacks a comparative assessment of the cross-sectional area of the nasal cavity or nasal obstruction (e.g., acoustic rhinometry). Second, comparative analyses with other techniques for caudal septal deviation were not performed. Third, this study was retrospective in nature.

## Conclusions

In conclusion, this study proved the efficacy and safety of the 3-D printed PCL mesh as a batten graft following the correction of caudal septal deviation using the wedge resection technique. Therefore, the 3-D printed PCL mesh could be a good alternative to the autograft for stably maintaining the modified septum in correction of caudal septal deviation.

## Data Availability

The datasets used and/or analysed during the current study are available from the corresponding author on reasonable request.

## Abbreviations

C&S technique: Cutting and suture technique
3-D: 3-Dimensional
PCL: Polycaprolactone
AR: Allergic rhinitis
NOSE: Nasal obstruction symptom evaluation
VAS: Visual analogue scale
